# Describing and comparing the characteristics of injured bicyclists and other injured road users: a prospective cohort study

**DOI:** 10.1186/s12889-016-2988-y

**Published:** 2016-04-14

**Authors:** Bamini Gopinath, Jagnoor Jagnoor, Ashley Craig, Annette Kifley, Michael Dinh, Rebecca Ivers, Soufiane Boufous, Ian D. Cameron

**Affiliations:** John Walsh Centre for Rehabilitation Research, Sydney Medical School, Kolling Medical Research Institute, University of Sydney, Sydney, Australia; The George Institute for Global Health, Sydney Medical School, University of Sydney, Sydney, Australia; Department of Trauma Services, Royal Prince Alfred Hospital, Sydney, Australia; Transport and Road Safety Research, The University of NSW, Sydney, Australia

**Keywords:** Bicyclist, Non-catastrophic injury, Socio-demographic, Cohort, Pre-injury

## Abstract

**Background:**

We aimed to establish the frequency and characteristics (e.g. socioeconomic, pre-injury, and crash-related parameters) of injured bicyclists and other injured road users.

**Methods:**

748 participants aged ≥17 years who had sustained a minor or non-catastrophic injury in a land-transport crash, were interviewed after presenting to a metro hospital emergency department in New South Wales, Australia. A telephone-administered questionnaire obtained information on socio-economic, pre-injury health, and crash-related characteristics. These factors were then compared between injured bicyclists and other road users (car driver/passengers, motorcyclists/pillion and pedestrians/skateboarders). Cycling injury severity was characterized by three metrics (sustaining multiple injuries; hospital admission for ≥12 h; and sustaining a head/neck and/or facial injury).

**Results:**

In this cohort of people with injuries, 238 (32 %) were bicyclists. Frequency of cycling injuries were significantly different between age-groups among men (*p* = 0.0002), and were more common in men aged 45–59. Bicyclists were more likely to be aged 45–59, married, have university/tertiary qualifications and have a professional occupation compared to other road users (all *p* <0.0001). Bicyclists compared to participants involved in other types of land transport crashes were more likely to self-report excellent general health (*p* = 0.01), and were less likely to report a great/overwhelming perceived danger of death or 15.0 % versus 23–41 %; *p* <0.0001). Frequency of upper extremity and lower extremity injuries in bicyclists were 81.9 % and 60.5 %, respectively. Explanatory variables significantly associated with injury severity metrics were age, education level, paid work status and perceived danger of death/disability in the crash.

**Conclusions:**

Minor cycling injuries were a relatively common cause of mild-moderate injury presentations to metro emergency departments. A wide spectrum of socio-demographic-, pre-injury-, and crash-related characteristics were related to cycling injuries.

## Background

Despite the personal and public health benefits, cycling is relatively risky compared to other forms of transport due to the fragility of the unprotected human body [1, 2]. In Australia, between 2000 and 2008, the estimated number of bicyclists increased by 36 % and the age-standardized rates of seriously injured bicyclists increased by 47 % (from 15.8 per 100,000 to 23.3 per 100,000) [1, 3]. Thus, it is likely that the number of crashes involving bicyclists could represent an emerging public health and policy issue. This is particularly pertinent, as third-party insurance schemes insure against motor vehicle crash injuries but do not cater for people in non-motorized vehicles, unless the bicyclist collides with a motor vehicle [1]. The economic and societal costs of these injuries are likely to be considerable due to their high incidence; and hence, there is a critical need to develop effective policy interventions aimed at reducing the overall burden attributable to cycling-related injuries [4, 5].

An Australian study of 313 adult bicyclists recruited from emergency departments reported that 52.0 % were injured in single-vehicle bicycle crashes. The remainder involved other road users: motor vehicles (20.8 %), other bicycles (18.8 %) and pedestrians (6.4 %) [1]. Over half of the cyclists (58.4 %) in that study had sustained a minor injury, while 36.1 % had a moderate injury. However, this study did not provide any data on the characteristics of cycling injuries. Data from an online survey of 2056 Australian cyclists showed that the incidence of cycling injuries over 1 year was 27 %, of which 49 % were minor injuries [4]. This study found that cycling frequency (days/week), cycling for competition and cyclists’ level of experience were significantly and inversely associated with an increased likelihood of reporting an injury [4]. Nonetheless, the above study is likely to be limited by sample selection bias due to the use of online survey data from members of a bicycle community and advocacy group [4]. More recently, *Dinh* et al. [6] showed that upper limb, head and facial injuries were the most common injuries encountered in a retrospective cohort of injured cyclists. Further, the use of bicycle helmets was associated with a significant reduction in odds of significant head injury. However, this was a small study (*n* = 258) at a single trauma center [6]. A recent Canadian study [7] found that about one-third of bicycle crashes were collisions with motor vehicles, and this was subsequently associated with more severe injuries than in other crash circumstances. Older age was also consistently associated with more severe injuries among Canadian bicyclists.

The objectives of the current epidemiological study include: 1) Establishing the frequency of minor cycling injuries sustained in a land-transport crash; 2) Describing and comparing the characteristics of injured bicyclists versus other injured road users (car driver/pillion, motorcyclists and pedestrians); and 3) Assessing the factors that were associated with cycling injury severity, as characterized by three metrics (sustaining multiple injuries; hospital admission for ≥12 h; and sustaining a head/neck and/or facial injury).

## Methods

### Study population

The study used an inception cohort design, and inception was defined as ‘within 28 days of injury.’ A cut-off time period of 28 days was chosen as this allowed sufficient time for recruitment and interviewing of participants, as well as being reasonably soon after the crash. The longitudinal follow-up of participants is currently underway, however, for the current report we present cross-sectional data obtained from the baseline survey. For the current report we use data that was collected from two major hospital emergency departments (ED) in central Sydney and surrounds; recruiting a total of 647 participants from both hospitals. In addition, 101 participants were identified from three EDs in rural NSW and two other suburban Sydney hospitals, that is, a total of 748 participants were analyzed for the current study. Research nurses at each hospital site screened the “First Net” ED database to identify potential participants.

Participants aged ≥17 years who had experienced a land transport crash resulting in a physical injury diagnosed by a medical practitioner in NSW between August 2013 and July 2014, were identified and invited to participate in the study. Detailed inclusion and exclusion criteria has been previously reported [8]. Briefly, inclusion study criteria were: a) injury due to motor vehicle crash diagnosed by a medical practitioner, or registered health practitioner, within 28 days of the crash; b) injury due to crash involving a motorized vehicle on land (public/private road/driveway/parking space or private/public land) in NSW; and c) injured person is a driver, rider, passenger, pillion passenger, pedestrian (person travelling on foot or operating toy vehicle, pedal car, barrow, billycart, non-motorized wheelchair or skateboard) or cyclist. Participants were excluded if they had: a) superficial injuries or injury due to a crash involving trains or light rail that are not covered by the compulsory third party (CTP) scheme; b) dementia or significant pre-existing cognitive impairment affecting ability to consent; c) sustained severe injuries (i.e. severe traumatic brain injury, spinal cord injury, extensive burns or multiple amputations), as these injuries are covered by the NSW Lifetime Care and Support Scheme and not by the CTP scheme; and d) minor isolated soft tissue injuries such as bruises, abrasions or cuts.

**Fig. 1:**
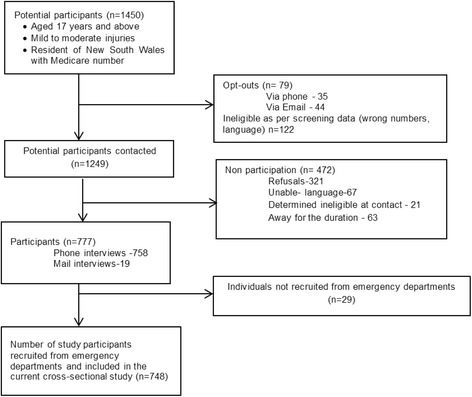
Flowchart of study participation

From the study sites, data for potential participants based on eligibility criteria were entered on a secure online platform, here called Research electronic data capture (REDCap) [9]. Once screened, potential participants were sent a letter that detailed the purpose of the study, what was involved and inviting them to participate in the study. Participants could opt-out of the study via telephone or through email. Participants who did not opt-out, within 1-week of the letter mail-out, were contacted by trained interviewers. Interviewers obtained informed consent by telephone and conducted the structured baseline interview. The interviews were conducted using Computer Aided Telephone Interview by trained interviewers. A total of 748 participants were recruited and surveyed at baseline, this included 238 bicyclists and 510 non-bicyclists. Flowchart of study participation is shown in Fig. 1. There were a small proportion of participants who were recruited from other sources e.g. insurance regulators claims database, general practitioners and physiotherapists. The study protocol was approved (including the verbal consent process) by a Sydney and South Western Sydney Local Health District Human Research Ethics Committee. This study was conducted according to the principles expressed in the Declaration of Helsinki.

### Assessing the characteristics of injured cyclists and other injured road users

Trained interviewers asked questions on socio-demographic variables including, age, sex, education level (university/tertiary or other), occupation, work status (in paid work or other), and marital status (married/defacto, divorced/widowed/separated or never married). Chronic illness was determined by asking participants if they had been diagnosed with any of the following: asthma, cancer, heart/circulatory condition, diabetes, mental and behavioral problems, and/or other in the last 3 months. Participants were asked to describe their general health status prior to the injury, using a 5-point Likert scale. Participants self-reported their smoking status i.e. whether they were current smokers or not. The 3-item Audit-C screen was administered to participants [10]. Scores on the Audit-C ranges from 0–12, and in general, the higher the score the more likely it is that the participant’s alcohol use is affecting his/her safety [10]. Body mass index (BMI) was calculated from self-reported height and weight, and classified according to WHO guidelines: <20 kg/m^2^ (underweight), 20–24.9 kg/m^2^ (normal), 25–29.9 kg/m^2^ (overweight), ≥30 kg/m^2^ (obese).

European Quality of Life-5 Dimensions (EQ-5D-3 L) scale was administered at baseline and was used to measure self-reported health-related quality of life pre-injury and post-injury [11]. In the current, study we focused on pre-injury EQ-5D-3 L measures. The first part of the EQ-5D-3 L has five dimensions: mobility, self-care, usual activities, pain/discomfort and anxiety/depression. Each dimension in the form of the measure used is divided into three levels: no problem, some problems and major problems. The second part is a 20-cm visual analogue scale (EQ VAS), which was modified slightly from the original version with a repetition of the question: ‘To help you say how good or bad your health state is, I have a scale in front of me (rather like a thermometer), on which the best health state you can imagine is marked 100 and the worst health state you can imagine is marked 0’ [11, 12].

Crash-related characteristics that were assessed in the study, included the participants’ self-perceived danger of death and/or disability (great or overwhelming; moderate; or small/none). Participants were also asked how many hours that they spent in hospital after the crash, and this was dichotomized as spending <12 h or ≥12 h in hospital. Whiplash injury was defined from self-reported current neck pain (derived from the Orebro Musculoskeletal Pain Screening Questionnaire) [13] at the time of the interview. Participants self-reported injury sites as well as any psychological injury sustained in the crash.

The study was not designed to focus on injury severity; hence, data was not collected on classical severity scoring using the Abbreviated Injury Scale. Hence, based on previous studies [7, 14] we classified injury severity using three metrics: 1) admitted to hospital ≥12 h; 2) sustaining a head/neck injury (includes facial injuries); and 3) sustaining multiple injuries i.e. two or more injuries and 4 or more injuries.

### Statistical analysis

Statistical analyses were performed using SAS v9.4. Baseline characteristics of bicyclists versus other road users in the cohort were summarized using descriptive statistics and differences in study characteristics were compared using the *χ*^2^-square test or *t*-tests where appropriate. Logistic regression analyses were also conducted to assess the association between each potential predictor (socio-demographic, pre-injury and crash-related characteristics) and each of the three metrics that classified cycling injury severity. Hence, a separate logistic regression model for each outcome and each possible predictive factor was constructed, and all data are presented as odds ratio (OR) and 95 % confidence intervals (CI). Significance level was set at *p* <0.05.

## Results

**Fig. 2:**
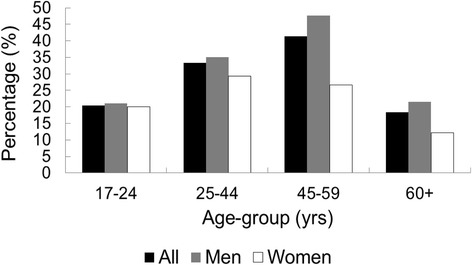
Age-sex distribution of bicyclists who sustained mild or moderate injuries at baseline

Of the 748 participants who were surveyed at baseline, and who had sustained mild or moderate injuries (i.e. non-catastrophic), 238 (32 %) were bicyclists and 510 (68 %) were non-bicyclists or other road users. Figure 2 shows the proportion of mild-moderate land transport injuries which involved bicyclists, by age and gender. Cycling was a more common injury source among 45–59 year old men (47.6 %) than in younger or older men (*p* = 0.0002), whereas the proportion with cycling-related injury did not differ significantly between age groups among women (*p* = 0.2).

Bicyclists were more likely to be aged 45–59, married, have university/tertiary qualifications and have a professional occupation compared to participants involved in other types of land transport crashes (Table 1). Bicyclists compared to the other three groups of land transport were less likely to smoke, be obese and had moderate alcohol intake. Bicyclists were also more likely to self-report excellent general health, and had higher EQ VAS and EQ-5D-3 L summary scores (except motorcyclists who had the same mean EQ-5D-3 L summary score) compared to participants injured in all other types of land transport crashes (Table 2). Bicyclists were less likely to report great or overwhelming perceived danger of either death or disability compared to all other groups (Table 2).

**Table 1:** Socio-demographic characteristics by crash type in the inception cohort

Characteristic	Bicyclist (*N* = 238)	Car driver passenger (*N* = 234)	Motorbike driver or passenger (*N* = 193)	Pedestrian/skateboard (*N* = 83)^a^	*P* value
Age (years)	41.7 (12.7)	43.1 (17.6)	38.5 (12.7)	42.6 (18.1)	0.01
Age group					<0.0001
17–24 years	22 (9.2)	42 (18.0)	28 (14.5)	14 (16.9)	
25–44 years	114 (47.9)	89 (38.0)	104 (54.9)	34 (41.0)	
45–59 years	84 (35.3)	54 (23.1)	48 (24.9)	17 (20.5)	
60+ years	18 (7.6)	49 (20.9)	13 (6.7)	18 (21.7)	
Gender					<0.0001
Male	180 (75.6)	113 (48.3)	168 (87.1)	50 (60.2)	
Female	58 (24.4)	121 (51.7)	25 (12.9)	33 (40.0)	
Educational level					<0.0001
University/tertiary	155 (65.1)	98 (41.9)	69 (36.0)	46 (55.4)	
Other	83 (34.9)	136 (58.1)	124 (64.3)	37 (44.6)	
Work status					<0.0001
Paid work	207 (87.0)	162 (69.2)	173 (89.6)	51 (61.5)	
Other	31 (13.0)	72 (30.8)	20 (10.4)	32 (38.6)	
Occupation (among paid workers)					0.0006
Professional	110 (53.7)	66 (41.0)	61 (36.1)	27 (52.9)	
Clerical services	7 (3.4)	12 (7.5)	9 (5.3)	2 (3.9)	
Technical/trade services	24 (11.7)	14 (8.7)	38 (22.5)	8 (15.7)	
Manager	35 (17.1)	17 (10.6)	27 (16.0)	6 (11.8)	
Community/personal services	7 (3.4)	18 (11.2)	8 (4.7)	3 (5.9)	
Labourer	6 (2.9)	9 (5.6)	11 (6.5)	2 (3.9)	
Sales worker	12 (5.8)	13 (8.1)	9 (5.3)	1 (2.0)	
Machinery operator or driver	4 (1.9)	12 (7.5)	6 (3.6)	2 (3.9)	
Marital status					0.002
Divorced, widowed or separated	8 (3.4)	21 (9.0)	9 (4.7)	6 (7.2)	
Married or defacto	132 (55.5)	113 (48.2)	91 (47.2)	26 (31.3)	
Never married	98 (41.2)	100 (42.7)	93 (48.2)	51 (61.5)	
No	231 (97.1)	212 (91.0)	184 (95.3)	78 (94.0)	
Moderate	31 (17.7)	47 (24.6)	37 (23.7)	11 (18.6)	
Small or none	125 (71.4)	88 (46.1)	79 (50.6)	36 (61.0)	

Data are presented as n (%) or mean (SD)

^a^Includes 68 pedestrians and 15 skateboard riders

**Table 2:** Pre-injury health, quality of life and crash-related characteristics by crash type in the inception cohort

Characteristic	Bicyclist (*N* = 238)	Car driver passenger (*N* = 234)	Motorbike driver or passenger (*N* = 193)	Pedestrian/skateboard (*N* = 83)^a^	*P* value
EQ5D VAS	86.9 (9.7)	83.9 (12.1)	86.1 (12.4)	85.6 (10.8)	0.03
EQ5D summary score	0.95 (0.10)	0.91 (0.14)	0.95 (0.09)	0.93 (0.13)	0.0001
BMI (from self-reported height and weight)	24.8 (3.8)	26.3 (6.0)	26.5 (4.1)	25.1 (5.7)	0.001
Self-reported health					<0.0001
Excellent	113 (47.5)	69 (29.5)	72 (37.3)	38 (45.8)	
Very good	90 (37.8)	91 (38.9)	79 (40.9)	21 (25.3)	
Good	31 (13.0)	51 (21.8)	35 (18.1)	16 (19.3)	
Fair	4 (1.7)	19 (8.1)	4 (2.1)	8 (9.6)	
Poor	0 (-)	4 (1.7)	3 (1.6)	0 (-)	
Current smoker					0.001
Yes	23 (9.7)	46 (19.7)	34 (17.6)	22 (26.5)	
No	215 (90.3)	188 (80.3)	159 (82.4)	61 (73.5)	
Alcohol intake – Audit c score					<0.0001
Zero	17 (7.1)	61 (26.1)	31 (16.1)	6 (7.2)	
1–3	78 (32.8)	88 (37.6)	60 (31.1)	25 (30.1)	
4–7	126 (52.9)	73 (31.2)	88 (45.6)	39 (47.0)	
8–12	17 (7.1)	12 (5.1)	14 (7.3)	13 (15.7)	
Any listed medical conditions on self-report					<0.0001
Yes	100 (42.0)	152 (65.2)	81 (42.0)	44 (53.0)	
No	138 (58.0)	81 (34.8)	112 (58.0)	39 (47.0)	
Obesity on self-report					0.03
Yes	7 (2.9)	21 (9.0)	9 (4.7)	5 (6.0)	
No	231 (97.1)	212 (91.0)	184 (95.3)	78 (94.0)	
Crash-related characteristics					
Perceived danger of death					<0.0001
Great or overwhelming	23 (10.0)	82 (35.7)	36 (19.0)	14 (17.1)	
Moderate	31 (13.4)	32 (13.9)	35 (18.4)	16 (19.5)	
Small or none	177 (76.6)	116 (50.4)	119 (62.6)	52 (63.4)	
Perceived danger of disability					<0.0001
Great or overwhelming	19 (10.9)	56 (29.3)	40 (25.6)	12 (20.3)	
Moderate	31 (17.7)	47 (24.6)	37 (23.7)	11 (18.6)	
Small or none	125 (71.4)	88 (46.1)	79 (50.6)	36 (61.0)	
Perceived danger of either death or disability					<0.0001
Great or overwhelming	26 (15.0)	79 (41.4)	52 (33.3)	14 (23.7)	
Moderate	34 (19.7)	41 (21.5)	36 (23.1)	13 (22.0)	
Small or none	113 (65.3)	71 (37.2)	68 (43.6)	32 (54.2)	

Data are presented as n (%) or mean (SD)

^a^Includes 68 pedestrians and 15 skateboard riders

Table 3 shows the nature of mild or moderate injuries sustained by bicyclists. Upper extremity injuries were more common than lower extremity injuries (81.9 % and 60.5 % respectively); with just over a third of injuries sustained to the head (37.8 %) or torso (35.3 %). The majority of bicyclists (77.7 %) had sustained injuries to multiple sites. More than one in three bicyclists were admitted to hospital for 12 h or more after the crash (Table 3).

**Table 3:** Nature of mild-moderate bicyclist injuries (*n* = 238)

Injury sites	N (%)
Head	90 (37.8)
Neck	37 (15.6)
Spine or back	44 (18.5)
Torso	84 (35.3)
Upper extremity	195 (81.9)
Lower extremity	144 (60.5)
Psychological injury	31 (13.0)
Multiple injury sites	185 (77.7)
Whiplash injury^a^	24 (10.1)
Hospital admission (≥12 h)	92 (38.7)

^a^Whiplash injury defined from current neck pain at time of interview

We also analyzed the associations between a range of factors that are associated with the various indicators of cycling injury severity (Table 4): a) being admitted to hospital (12 h or more); b) injury to head/neck (includes facial injuries); c) multiple injuries (2 or more); and d) multiple injuries (4 or more). Bicyclists aged 17–24 were 73 % less likely to have an injury to head/neck, while those aged 45–59 were 96 % more likely to be in hospital for 12 h or more following the injury as well as twice as likely to have multiple injuries (≥4 injuries) (Table 4). Being tertiary educated and being in paid work status was positively associated with sustaining ≥2 and ≥4 injuries, respectively. Self-reported obesity was associated with 80 % reduced odds of sustaining multiple injuries (≥2). Self-perceived danger of death was associated with hospital admission status and sustaining four or more injuries (Table 4). Crash circumstances e.g. whether the cyclist was solely involved (reference group) in the crash or whether it involved a motor vehicle, pedestrian, another cyclist etc. was not associated with any of the injury severity indices (data not shown).

**Table 4:** Associations between different factors and cycling injury severity indicators (separate model for each outcome and each possible explanatory factor), presented as unadjusted odds ratio (OR) and 95 % confidence interval (CI)

	Hospital admission (>12 h)	Injury to head or neck (includes facial injuries)	Multiple injuries (≥2)	≥4 injuries
Demographics				
Age group				
17–24 years	1.56 (0.61, 3.99)	**0.27 (0.09, 0.79)**	0.57 (0.20, 1.56)	0.57 (0.12, 2.67)
25–44 years	1.0 (ref)	1.0 (ref)	1.0 (ref)	1.0 (ref)
45–59 years	**1.96 (1.09, 3.51)**	0.67 (0.38, 1.18)	0.85 (0.43, 1.68)	**2.15 (1.06, 4.35)**
60+ years	2.26 (0.82, 6.17)	0.93 (0.34, 2.52)	2.13 (0.46, 9.92)	2.20 (0.69, 6.95)
Gender				
Male	1.04 (0.56, 1.92)	0.71 (0.39, 1.29)	1.86 (0.95, 3.64)	0.93 (0.44, 1.93)
Female	1.0 (ref)	1.0 (ref)	1.0 (ref)	1.0 (ref)
Educational level				
University/tertiary	0.93 (0.54, 1.61)	1.53 (0.89, 2.64)	**1.95 (1.05, 3.64)**	**2.29 (1.07, 4.88)**
Other	1.0 (ref)	1.0 (ref)	1.0 (ref)	1.0 (ref)
Work status				
Paid work, including self-employment	0.99 (0.46, 2.17)	1.89 (0.85, 4.21)	1.25 (0.52, 2.99)	**8.57 (1.13, 64.5)**
Other	1.0 (ref)	1.0 (ref)	1.0 (ref)	1.0 (ref)
Marital status				
Divorced, widowed/separated	2.75 (0.62, 12.2)	1.81 (0.41, 7.99)	1.08 (0.20, 5.71)	1.71 (0.31, 9.24)
Married/defacto	1.01 (0.58, 1.72)	0.80 (0.47, 1.35)	1.55 (0.83, 2.89)	1.44 (0.73, 2.84)
Never married	1.0 (ref)	1.0 (ref)	1.0 (ref)	1.0 (ref)
Pre-injury health and QOL				
EQ5D VAS	1.02 (0.98, 1.05)	1.00 (0.97, 1.03)	0.99 (0.96, 1.03)	0.99 (0.96, 1.02)
EQ5D summary score	0.32 (0.02, 4.58)	2.08 (0.14, 30.1)	1.51 (0.07, 33.6)	0.93 (0.03, 24.9)
EQ5D – any issues reported on subscales	1.21 (0.66, 2.21)	0.76 (0.42, 1.40)	0.84 (0.42, 1.70)	1.11 (0.53, 2.32)
Self-reported health status				
Excellent	1.0 (ref)	1.0 (ref)	1.0 (ref)	1.0 (ref)
Very good	0.69 (0.39, 1.22)	1.12 (0.64, 1.94)	1.01 (0.51, 1.99)	1.24 (0.62, 2.51)
Good	0.72 (0.31, 1.64)	0.67 (0.29, 1.52)	0.57 (0.23, 1.36)	1.36 (0.51, 3.58)
Fair	0.43 (0.04, 4.32)	3.65 (0.36, 36.1)	-	1.55 (0.15, 15.7)
Poor	-	-	-	-
Current smoker				
Yes	0.41 (0.14, 1.14)	0.92 (0.38, 2.18)	1.03 (0.36, 2.93)	0.84 (0.27, 2.60)
No	1.0 (ref)	1.0 (ref)	1.0 (ref)	1.0 (ref)
Alcohol intake – Audit c score				
Zero	1.0 (ref)	1.0 (ref)	1.0 (ref)	1.0 (ref)
1–3	0.87 (0.30, 2.49)	0.96 (0.33, 2.76)	1.03 (0.30, 3.54)	0.71 (0.20, 2.51)
4–7	0.63 (0.22, 1.73)	0.87 (0.31, 2.41)	1.13 (0.34, 3.74)	0.85 (0.25, 2.81)
8–12	0.47 (0.11, 1.93)	1.27 (0.33, 4.87)	1.00 (0.20, 4.88)	0.70 (0.13, 3.72)
Medical conditions on self-report				
Yes	1.03 (0.61, 1.74)	1.20 (0.71, 2.01)	0.84 (0.45, 1.56)	0.83 (0.42, 1.59)
No	1.0 (ref)	1.0 (ref)	1.0 (ref)	1.0 (ref)
Obesity on self-report				
Yes	1.20 (0.26, 5.47)	0.19 (0.02, 1.63)	**0.20 (0.04, 0.93)**	0.67 (0.07, 5.71)
No	1.0 (ref)	1.0 (ref)	1.0 (ref)	1.0 (ref)
Overweight (BMI > =25)				
Yes	0.79 (0.46, 1.36)	0.75 (0.43, 1.27)	0.91 (0.48, 1.73)	1.49 (0.77, 2.87)
No	1.0 (ref)	1.0 (ref)	1.0 (ref)	1.0 (ref)
Crash-related characteristics				
Perceived danger of death				
Great or overwhelming	2.07 (0.86, 4.98)	1.28 (0.53, 3.05)	2.34 (0.66, 8.25)	2.54 (0.95, 6.78)
Moderate	1.37 (0.63, 2.99)	1.93 (0.88, 4.18)	2.37 (0.78, 7.14)	**3.19 (1.37, 7.44)**
Small or none	1.0 (ref)	1.0 (ref)	1.0 (ref)	1.0 (ref)
Perceived danger of disability				
Great or overwhelming	**2.82 (1.05, 7.54)**	2.00 (0.75, 5.31)	5.68 (0.73, 44.4)	**4.05 (1.44, 11.4)**
Moderate	1.13 (0.49, 2.57)	1.76 (0.80, 3.89)	2.13 (0.69, 6.58)	1.34 (0.48, 3.70)
Small or none	1.0 (ref)	1.0 (ref)	1.0 (ref)	1.0 (ref)
Accident circumstances ^a^				
Cyclist alone	1.0 (ref)	1.0 (ref)	1.0 (ref)	1.0 (ref)
Collision involving a motor vehicle	0.81 (0.41, 1.59)	0.58 (0.30, 1.13)	1.56 (0.69, 3.49)	1.32 (0.59, 2.97)
Collision with a cyclist, person or animal	1.02 (0.27, 3.84)	4.07 (0.82, 20.1)	3.11 (0.37, 25.7)	3.16 (0.81, 12.3)
Games or stunts, cycling events, and mountain biking	0.88 (0.34, 2.27)	0.58 (0.22, 1.50)	0.92 (0.32, 2.59)	0.75 (0.20, 2.78)

Bolded figures indicate significant estimates

^a^Accident circumstances were assessed from participant comments prompted by open interviewer questions at wave 2. ‘Cyclist alone’ includes averted collisions, bike failure or malfunction, errors, falls, slips and slides, hitting potholes or obstructions or other inanimate objects, wet or greasy roads. ‘Collision involving a motor vehicle’ includes collisions with either a moving or stationary car, or a suddenly opened car door

## Discussion

We observed that a wide-spectrum of socio-economic, pre-injury and crash-related factors were associated with minor injuries in cyclists compared to other road users. Moreover, socio-demographic variables and crash-related characteristic were also independently associated with injury severity metrics among bicyclists.

Underreporting of injuries from road traffic crash is a common problem for all transport modes, but it is most extensive for bicycling [5]. The frequency of cycling injuries (32 %) observed in this cohort is similar to the rate reported in a recent NSW study, which showed that 30 % of road trauma presentations to EDs in Sydney are due to cycling-related injuries [15]. Understanding the demographic characteristics of cycling injuries could assist in planning targeted interventions to improve cycling safety [4]. Although a prior Australian study found no sex differences in cycling injury incidence [4], our findings concur with other published research that shows a substantially higher proportion of non-catastrophic cycling injuries involved men rather than women [6, 16, 17]. This is consistent with an Australian study which showed that males are more likely to participate in cycling than females i.e. 20.9 % of males and 12.4 % of females had ridden in the previous week [1]. Alternatively, a greater propensity for risk-taking and speed may provide more opportunities for male cyclists to be involved in land-transport crashes [7].

It has been previously suggested that the willingness to walk or bicycle for short trips, instead of using motorized vehicles, increases with higher education levels, suggesting a possible socioeconomic gradient in utility cycling [4]. Our findings are in agreement with this suggestion, given that bicyclists compared to non-bicyclists (car driver, motorcyclists, and pedestrians) who had sustained non-catastrophic injuries in the crash were more likely to have a higher education level (tertiary qualified), and be in paid employment. Moreover, over half of the bicyclists in our cohort were professionals compared to 41 % of car drivers/passengers and 36 % of motorcyclists/pillion passengers. However, we would like to advise caution in interpreting this result as we do not have a representative sample of bicyclists i.e. we have only capture those cyclists who had sustained a non-catastrophic injury and had presented to a hospital ED.

Around 82 % of cyclists had sustained an upper extremity injury in our cohort; and this concurs with other epidemiological studies that have shown injuries to the upper limbs is particularly frequent among bicyclists [6, 14, 17–20]. In contrast, just over a third of bicyclists had sustained head injuries in our study, which was similar to a head injury rate of 34.7 % observed among bicyclists in a USA study [14]. Interestingly, around a third of bicyclists reported being in hospital for 12 or more hours. This could be due to a high frequency of multiple injury sites (78 %) being observed in this cohort, which could have led to the medical practitioner ensuring that the bicyclist is appropriately assessed and treated before being sent home.

Pre-injury health measures were significantly different between bicyclists and non-bicyclists in this cohort. Cycling for transport or as a leisure time activity has been associated with significantly higher general life satisfaction and sense of personal wellbeing [21]. Our study adds to this evidence-base by showing that bicyclists compared to persons involved in other types of land transport crashes had significantly higher EQ-5D-3 L summary scores (except for motorcyclists who had the same mean EQ-5D-3 L summary score) and EQ-5D-3 L VAS scores prior to the injury. Bicyclists versus persons involved in all other crash types also had a lower frequency of modifiable lifestyle risk factors prior to the injury, that is, a significantly lower frequency of smoking, obesity and other medical conditions or comorbidities, which could also contribute to their overall sense of wellbeing.

Injury severity has been proposed as a second and equally important criterion that can be used by the lay public to evaluate the apparent safety of cycling [7]. Not surprisingly, we observed age to be significantly associated with several of the injury severity metrics (being hospitalized for >12 h and sustaining 4 or more injuries). Cyclists aged 45–59 years had the highest odds of sustaining a severe injury. Although, increased odds were observed for those aged 60+, it was non-significant. It is likely that smaller numbers in this age-group and resulting lack of statistical power led to the inability to detect modest associations with injury severity. The published literature [4, 7, 14, 22–24] also shows that age is significantly associated with cycling injury severity, this is possibly explained by decreasing physical robustness and comorbidities (e.g. osteoarthritis, cardiovascular and respiratory diseases) with age [7]. Hence, programs that allow older cyclists to better understand the impact of their greater fragility could be beneficial as it could prompt older adults to adjust their cycling behavior to reduce their probability of more severe injury should they be in a crash [23]. Finally, the relationship between perceived danger of death/disability in the crash and severity of cycling injury observed in our study is likely due to the fact that participants are reporting on crash characteristics after the injury, hence, those cyclists who had multiple injuries are the ones who are likely to perceive a great/overwhelming danger of death or disability.

Our findings contribute to the debate as to whether bicyclists involved in any road trauma should be included in the third party insurance scheme. Specifically, it could serve as a strategy to improve helmet usage and other safety gear, that is, reductions in insurance payout if the bicyclist was not wearing a helmet. Cycling campaigns, public education and appropriate cycling infrastructure (e.g. cycling lanes/paths) is vital in contributing to a reduction in injuries [18], with our findings suggesting that targeting middle-aged men through such campaigns could contribute to potential reductions in non-catastrophic cycling injuries. Further, since upper extremity and torso injuries were also relatively frequent in our cohort, this suggests that bicyclists might receive additional protection through wearing gear such as rigid protective vests and extremity guards (e.g. wrist or elbow guards) to minimize injury to the torso (e.g., rib fractures, shoulder dislocations) and to the extremities (e.g. wrist fractures) [20].

Key strengths of this study include its recruitment strategy and the collection of data on a wide range of parameters associated with minor cycling injuries. Study limitations also deserve discussion. Our study does not provide prevalence-based estimates of overall rates of cycling injury, and we were not able to include bicyclists who did not present to an ED or whose injury was not diagnosed by a registered medical practitioner. Future research is therefore needed to estimate the true overall burden of cycling injuries. We did not collect data on the actual location or circumstance of injury (e.g. roadway or junction, on a shared path), cycling intensity (recreational or transport cyclists, monthly, weekday/weekend, night/day), the interactions involved (whether the cycling collision involved a motor vehicle, another bicycle or pedestrian etc.), and other hospitalization details (e.g. procedures undergone in hospital). We present only cross-sectional data in the current report, however, this study is ongoing and it is anticipated that prospective data will be available in near future. Therefore, it will be of interest to report longer term health outcomes after cycling injuries in this cohort in further studies.

## Conclusions

From a public health perspective, the costs associated with caring for injured cyclists are likely to be significant as cycling becomes an increasingly popular and environmentally friendly form of transport. This descriptive study shows that presentation of cycling injuries to metro hospital EDs were relatively common. Non-catastrophic cycling injuries were characterized by a wide spectrum of demographic and health-related characteristics, and some of these factors could be potentially amenable to public health interventions. Socio-demographic and crash-related factors were also significantly associated with several injury severity metrics. Our study findings could have potential implications for future implementation of safety measures to reduce the morbidity and burden associated with cycling injuries.

### Ethics approval and consent to participate

The study protocol was approved (including the verbal consent process) by a Sydney and South Western Sydney Local Health District Human Research Ethics Committee. All participants have provided consent to participate in this study. This study was conducted according to the principles expressed in the Declaration of Helsinki.

### Consent to publish

Not applicable.

### Availability of data and materials

Given that this is an ongoing study we will not share the data at this stage, however, the dataset would be made publicly available once the study is completed at the end of this year. The corresponding author can be contacted for any specific queries on the data that was analyzed for this report.

## Abbreviations

BMI: body mass index
CTP: compulsory third party
ED: emergency department
NSW: New South Wales
VAS: visual analogue scale
